# The Influence of Nitrogen on Culturable Phyllosphere Microorganisms and the Incidence of *Botrytis cinerea* in Postharvest Leafy Vegetables

**DOI:** 10.3390/jof11110787

**Published:** 2025-11-02

**Authors:** Viktorija Vaštakaitė-Kairienė, Darius Jermala, Alma Valiuškaitė, Kristina Bunevičienė, Armina Morkeliūnė, Neringa Rasiukevičiūtė

**Affiliations:** Lithuanian Research Centre for Agriculture and Forestry, Instituto al. 1, LT-58344 Akademija, Lithuania; viktorija.vastakaite-kairiene@lammc.lt (V.V.-K.); darius.jermala@lammc.lt (D.J.); alma.valiuskaite@lammc.lt (A.V.); kristina.buneviciene@lammc.lt (K.B.); armina.morkeliune@lammc.lt (A.M.)

**Keywords:** basil, lettuce, pak choi, postharvest, postharvest pathology

## Abstract

Lettuce (*Lactuca sativa*), pak choi (*Brassica rapa*), and basil (*Ocimum basilicum*) were grown in hydroponic NFT systems under four nitrate levels (80–180 mg L^−1^ N). We measured natural microbial contamination by plating nutrient-solution samples and leaf washes to obtain colony-forming unit (CFU) counts of bacteria and fungi. Separately, postharvest leaves were artificially inoculated with *Botrytis cinerea* and stored at 22 °C or 4 °C for 7 days to assess gray mold. In lettuce, high N (180 mg L^−1^) markedly increased culturable microbial loads in both solution and leaves, whereas pak choi microbial counts remained low at all N levels. Basil showed a non-linear response: CFU counts peaked at moderate N (120 mg L^−1^) and were lower at both deficit and excess N. At 22 °C, gray mold severity in pak choi increased with N; leaves fertilized at N150–180 suffered about 1.5–2 times greater lesion area than those at N80. By contrast, lettuce exhibited the worst decay under N deficiency: N80 leaves developed the largest lesions by 4–7 DPI, while moderate N (120–150 mg L^−1^) minimized disease progression. Basil was highly susceptible under warm storage: all N levels reached near-total decay by 7 days, though N120 delayed early infection slightly. Refrigeration (4 °C) greatly suppressed lesion development in lettuce and pak choi, although high-N pak choi still showed ~20–30% more infected area than low-N after 7 days. Basil, however, suffered chilling injury at 4 °C, and all refrigerated basil leaves decayed severely (regardless of N). These results indicate crop-specific nutrient and storage strategies: avoid excessive N in pak choi, maintain balanced N in lettuce, and handle basil with non-chilling methods to reduce postharvest gray mold.

## 1. Introduction

Leafy vegetables are nutrient-rich crops that provide essential vitamins, minerals, fiber, and phytochemicals beneficial to human health [1,2]. Ensuring a sufficient supply of these vegetables is important for food security, yet they are highly perishable and can spoil rapidly after harvest [3]. Poor postharvest handling and storage can result in losses of up to 50% of leafy vegetable yields [4]. Such losses not only diminish the availability of nutritious food but also represent a significant economic setback for producers.

Microbial activity on leaf surfaces is a major factor in the spoilage of vegetables during storage. The above-ground parts of plants (the phyllosphere) harbor diverse communities of bacteria and fungi that are generally benign or even beneficial to plant health and crop quality before harvest [5]. When leaves are damaged or begin to senesce, however, some of these phyllosphere microorganisms can proliferate rapidly and accelerate spoilage by invading tissues and triggering plant stress responses [5,6]. Improving our understanding of these plant–microbe interactions is crucial for developing strategies to mitigate postharvest spoilage [6]. Notably, the diversity and ecological roles of fungal communities on fresh vegetables remain largely unexplored [5].

For many crops, postharvest fungal diseases cause substantial losses, often exceeding those incurred during cultivation [7]. Among these pathogens, *Botrytis* species—especially *B. cinerea*, the causal agent of gray mold—are particularly destructive in horticultural produce, including leafy greens [8]. *B. cinerea* is a necrotrophic fungus that often infects host tissues through wounds incurred during harvesting and handling [8]. The pathogen can remain latent on the plant until favorable conditions allow it to proliferate [8]. Under storage conditions, it produces characteristic gray conidial masses on affected leaves and readily spreads to adjacent produce through airborne spores and mycelial growth [8]. The management of *B. cinerea* in postharvest storage relies largely on fungicides, but concerns about chemical residues and regulatory restrictions have led to increased demand for alternative control methods [9].

Considering these challenges, plant nutritional strategies are being explored as a means to enhance crop disease resistance. In particular, the supply of nitrogen (N) has a strong influence on plant growth and can affect interactions with pathogens. Excessive N fertilization has been linked to increased severity of several foliar diseases, including downy mildew, powdery mildew, rusts, stem rot, and rice blast [10,11,12,13]. High N availability promotes the growth of lush, tender plant tissues and can alter plant defense mechanisms; these changes create conditions that are often favorable for pathogen infection [10,11,12,13]. The relationship between N availability and disease outcomes, however, is complex and can depend on the specific plant–pathogen system, highlighting the need for further research into how N influences plant immunity and microbial dynamics.

However, the influence of N fertilization on culturable phyllosphere microbial loads and on the development of postharvest fungal diseases in leafy vegetables remains largely unexplored. In this study, we hypothesized that higher N concentrations in the growing medium would alter the culturable phyllosphere fungal community as measured by plate counts and thereby affect the incidence of *B. cinerea* after harvest. Indeed, recent studies show that N fertilization can strongly reshape phyllosphere microbial communities and disease outcomes. For instance, Wu et al. [14] observed that high N shifts in rice markedly increased pathogenic fungal abundance on leaves, whereas moderate N enrichment benefited beneficial microbes and minimized pathogen levels. Similarly, Ouhibi et al. [15] reported that lettuce grown under low N was more resistant to *B. cinerea* than high-N lettuce, consistent with the idea that elevated N can favor gray mold development. In this study, we hypothesized that higher N concentrations in the growing medium would alter the phyllosphere fungal community and, in turn, affect the incidence of *B. cinerea* during postharvest storage. To test this hypothesis, we conducted experiments on three widely cultivated leafy vegetables—lettuce (*Lactuca sativa*), basil (*Ocimum basilicum*), and Chinese cabbage (*Brassica rapa*)—grown under different N regimes and evaluated changes in their phyllosphere fungal communities and the incidence of *B. cinerea* after harvest. Our objective was to determine whether varying N fertilization can modulate the phyllosphere microbial loads and thereby affect postharvest disease development in leafy vegetables. In our experimental design, we explicitly test this hypothesis by measuring culturable phyllosphere fungi and bacteria in hydroponic solution and on leaves at harvest and at the end of postharvest storage. We compared gray mold incidence under 80–150 N treatments, thereby evaluating the influence of N supply on *B. cinerea* in postharvest leafy vegetables.

## 2. Materials and Methods

### 2.1. Growing Conditions

The experiments were performed at the Lithuanian Research Centre for Agriculture and Forestry (LAMMC), Institute of Horticulture (IH), in the greenhouse from May to September 2024. The seeds of lettuce (*Lactuca sativa* L., “Bionda A Foglia Riccia”), basil (*Ocimum basilicum* L., “Italiano Classico”), and Chinese cabbage (*Brassica rapa* L., “Arax”) were sown into rock wool cubes (2.5 × 2.5 cm) and pre-soaked in deionized water. Seeded cubes were placed in plastic trays and covered with agrotextile film. After four days, trays were uncovered, and deionized water was exchanged with a modified Hoagland nutrient solution containing the following average nutrient concentrations (mg L^−1^): P, 25; K, 100; Ca, 80; Mg, 40; S, 53; Fe, 2; Mn, 0.1; Cu, 0.1; B, 0.2; and Zn, 1. The pH was 5.5–6.5, and the electrical conductivity (EC) was 0.13–0.17 S m^−1^ (GroLine HI9814, Hanna Instruments, Woonsocket, RI, USA). After 10 days, seedlings with the first pair of true leaves were transplanted to vertical nutrient film technique (NFT) hydroponic systems with nutrient solutions with different N concentrations (Table 1). The concentrations of N were increased by adding ammonium nitrate to the solution.

### 2.2. Natural Microbial Contamination of Fungi and Bacteria in Hydroponic Solution

At harvest, 50 mL of nutrient solution was collected per replicate. No intentional inoculation of the hydroponic nutrient solution was performed; the detected microbial counts represent naturally occurring fungal and bacterial contamination present in the system. Then, suspension was diluted from a 10^−1^ to 10^−3^ series by the serial dilution method. The 0.1 mL diluent was spread plated on PDA. The samples were Petri incubated in an incubator at 22 ± 2 °C and about 60% relative humidity in the dark. The mean total count of fungi and bacteria in the nutrient solution was counted in units and calculated to CFU mL^−1^. Colonies counted by colony counter (SC6 Plus, Stuart Equipment, Cole-Parmer Ltd., Stone, Staffordshire, UK). The CFU was assessed according to the standard ISO 7218:2007 [16]. The colony count measurements were as follows: (a) harvest—evaluated after 2, 4, and 7 days post-inoculation (DPI); (b) end of postharvest storage—evaluated after 2 DPIs.

### 2.3. Natural Microbial Contamination of Fungi and Bacteria on Leafy Vegetables Leaves at Harvest and During Postharvest Storage

At harvest or the end of postharvest, 5 leaves per replicate were randomly collected and transferred to 90 mL sterile deionized water (SDW) for 10 min to homogenize the sample. Then, suspension was diluted from a 10^−1^ to 10^−3^ series by the serial dilution method. The 0.1 mL diluent was spread plated on potato dextrose agar (PDA) (Liofilchem, Roseto degli Abruzzi, Italy). The samples were Petri incubated in an incubator at 22 ± 2 °C and about 60% relative humidity in the dark. Natural microbial contamination on vegetable leaves was measured in Colony Forming Units (CFUs). The mean total count of fungi and bacteria on the leaves was evaluated by mean colony counts, diluted and enumerated, and estimated for the CFU per milliliter sample (CFU mL^−1^). Colonies were counted by colony counter (SC6 Plus, Stuart Equipment, Cole-Parmer Ltd., Stone, Staffordshire, UK). The CFU was assessed according to the standard ISO 7218:2007 [16]. The natural microbial contamination of harvested plants was assessed to determine the background levels of naturally occurring microorganisms. CFU were evaluated at 2, 4, and 7 days after harvest. In addition, natural contamination of leafy vegetables was analyzed at the end of postharvest storage, where CFU were determined after 2 days of incubation. Types of fungal pathogens were determined at 7 DPIs, visually and microscopically, based on typical colonies’ morphological and cultural characteristics [17,18,19,20].

### 2.4. Artificial Infection with B. cinerea of Leafy Vegetables Leaves

To evaluate the impact of mineral nutrition on *B. cinerea* incidence and lesion development, artificial infection was performed. A single-spore *B. cinerea* isolate (accession LT11B_BRA_189) from the LAMMC Institute of Horticulture, Laboratory of Plant Protection culture collection was used. The isolate was stored on potato dextrose agar (PDA) slants at 4 °C and subcultured on PDA at 22 ± 2 °C for 7 days prior to inoculation. Randomly selected leaves were used for inoculation. Leaves were put on filter paper in a sterile Petri dish, and 5 mL of sterile deionized water was added. The 6 mm disk (mycelial side down) of each isolate was placed in the center of the leaf. A total of 12 leaves per replicate were used in the treatment. Plates were incubated at 22 ± 2 °C and 4 ± 2 °C temperatures in darkness. Resistance was assessed at 2, 4, and 7 DPIs. The whole leaf area and diameter of infection (mm) were measured. Disease incidence per leaf area was calculated as [21]:

$$Leaf\;damage\;\left(\%\right)=\frac{lesion\;area}{leaf\;area}\times 100\%$$

### 2.5. Statistical Analysis

For statistical analysis, data were processed using the XLSTAT software (2025.1, Addinsoft, Paris, France), using one-way analysis of variance, or ANOVA, and Tukey’s HSD test at a confidence level of *p* < 0.05.

## 3. Results

### 3.1. Natural Fungal and Bacterial Contamination in Hydroponic Solutions

**Figure 1:**
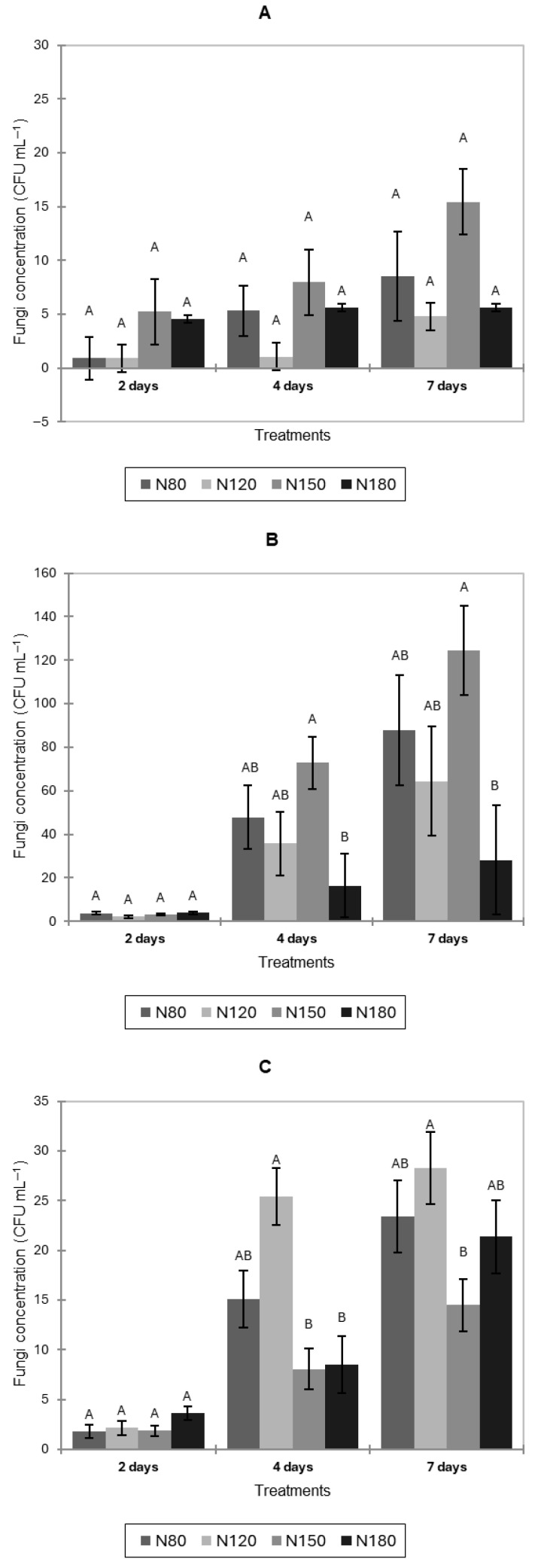
Natural fungal contamination (CFU mL^−1^) in hydroponic nutrient solutions of pak choi (**A**), lettuce (**B**), and basil (**C**) after 2, 4, and 7 days of cultivation under four nitrogen levels (N80, N120, N150, and N180). Letters indicate significant differences among treatments (*p* < 0.05). Data is presented as the mean of twelve replicates (n = 12). Error bars indicate standard error.

Microbial loads reported in this section reflect natural contamination in the hydroponic solutions under different nitrogen levels; no inoculation was applied to the nutrient media. Natural fungal counts in the hydroponic solution of pak choi increased consistently over time across all nitrogen treatments; however, no significant differences were observed among treatments at any sampling point (*p* > 0.05) (Figure 1A).

In lettuce, fungal contamination in the nutrient solution also increased over time under all nitrogen treatments (Figure 1B). After 2 days, all treatments exhibited similarly low fungal levels, suggesting minimal initial contamination. At 4 and 7 days, N180 showed significantly lower fungal concentrations than N150 (*p* < 0.05).

Fungal concentrations in the nutrient solution of basil increased from 2 to 7 days under all nitrogen treatments (Figure 1C). At 4 days, N120 had significantly higher fungal concentrations than N150 and N180 (*p* < 0.05). By 7 days, N120 again showed significantly higher fungal levels than N150.

**Figure 2:**
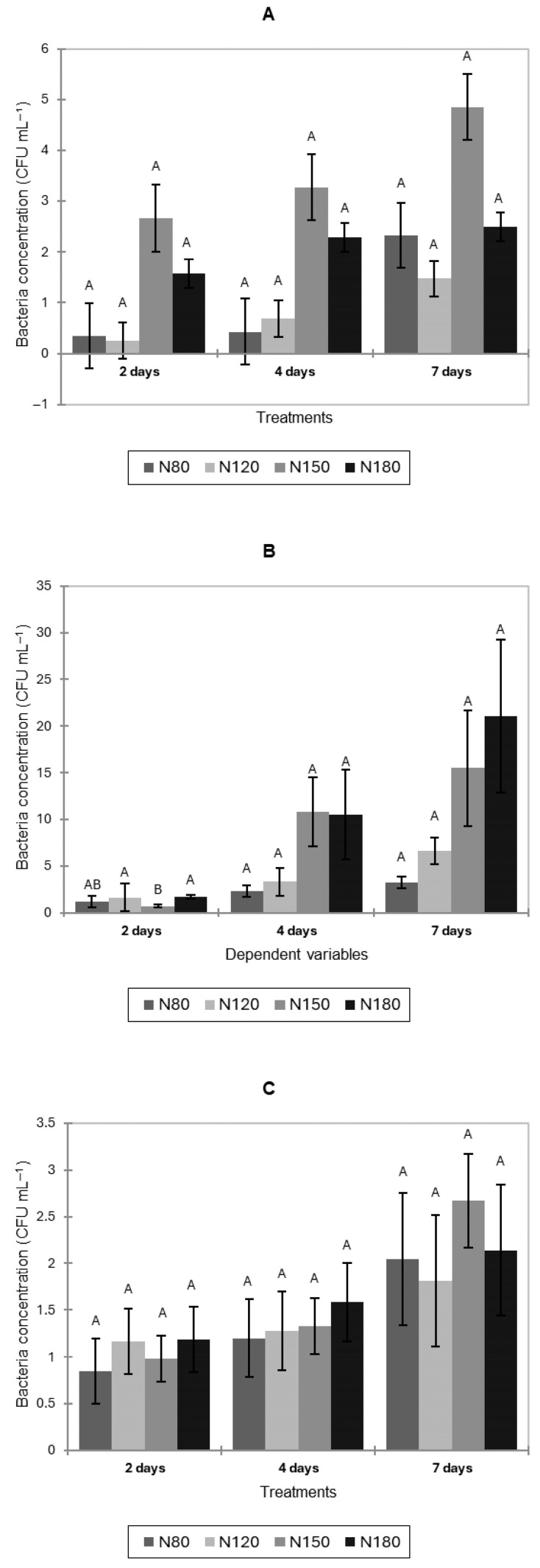
Natural bacterial contamination (CFU mL^−1^) in hydroponic nutrient solutions of pak choi (**A**), lettuce (**B**), and basil (**C**) after 2, 4, and 7 days of cultivation under four nitrogen levels (N80, N120, N150, and N180). Letters indicate significant differences among treatments (*p* < 0.05). Data is presented as the mean of twelve replicates (n = 12). Error bars indicate standard error.

Bacterial concentrations in the hydroponic solution of pak choi increased over time under all nitrogen treatments (Figure 2A). No significant differences among nitrogen levels were detected at any sampling point (*p* > 0.05).

Bacterial concentrations in the hydroponic solution of lettuce increased over time under all nitrogen treatments (Figure 2B). At 2 days, N150 showed significantly lower bacterial concentrations than N120 and N180. No significant differences among treatments were observed after 4 and 7 days.

For basil, bacterial concentrations in the nutrient solution also increased over time under all nitrogen treatments (Figure 2C). Although slight variations were observed after 2 days, no significant differences occurred among treatments at any sampling point.

### 3.2. Natural Fungal and Bacterial Contamination of Leafy Vegetables Leaves

**Figure 3:**
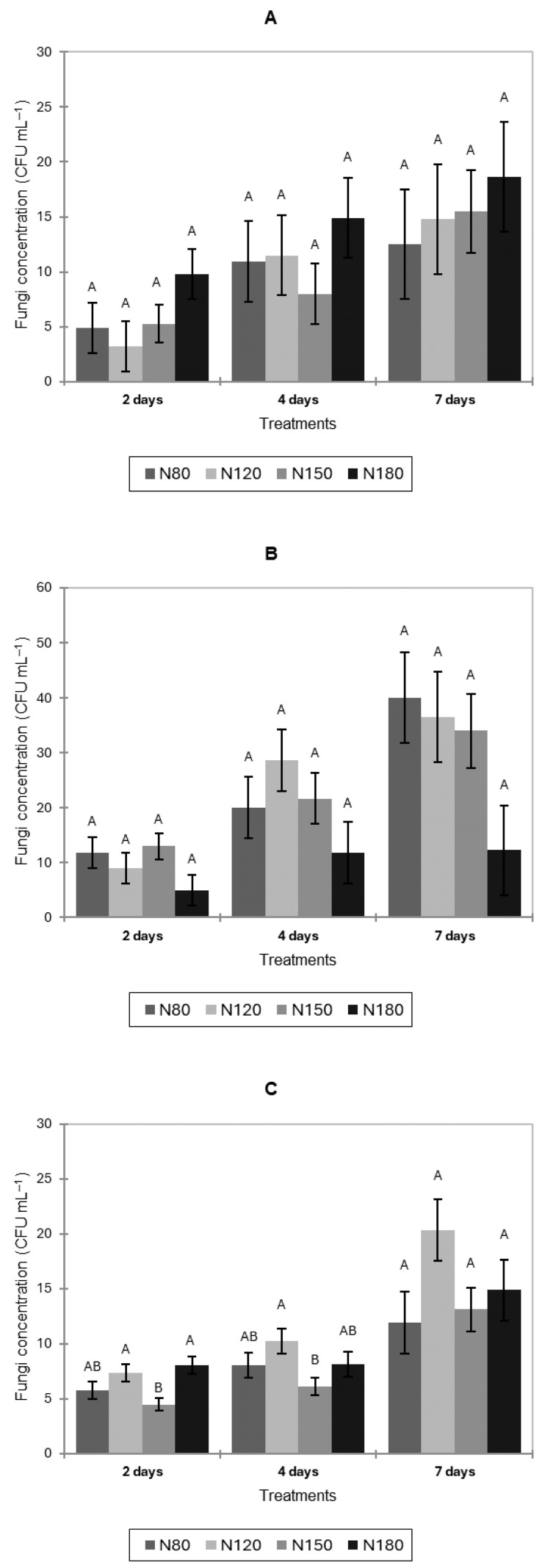
Natural fungal contamination (CFU mL^−1^) of pak choi (**A**), lettuce (**B**), and basil (**C**) leaves after 2, 4, and 7 days of cultivation under four nitrogen levels (N80, N120, N150, and N180). Letters indicate significant differences among treatments (*p* < 0.05). Data is presented as the mean of twelve replicates (n = 12). Error bars indicate standard error.

Natural fungal contamination on pak choi and lettuce leaves increased over time under all nitrogen treatments, with no significant differences among treatments (Figure 3A,B). In basil (Figure 3C), N150 showed significantly lower fungal counts than N120 and N180 after 2 days, and significantly lower fungal abundance than N120 after 4 days. No significant differences were observed at 7 days.

**Figure 4:**
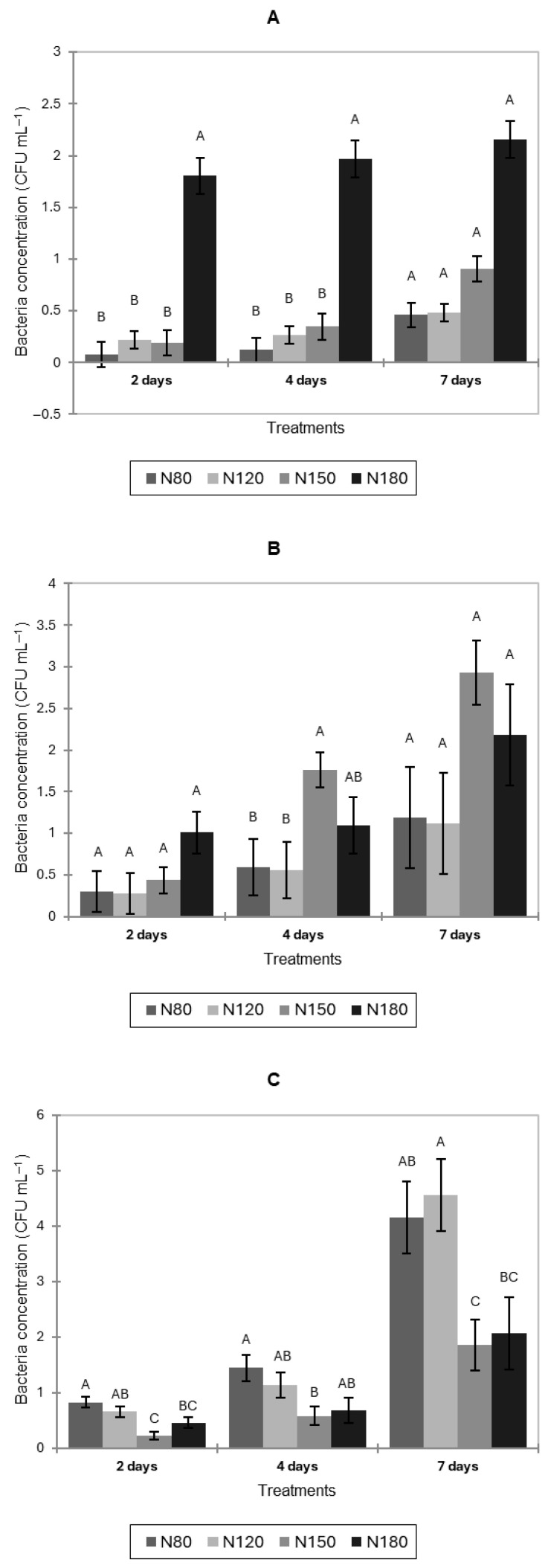
Natural bacterial contamination (CFU mL^−1^) of pak choi (**A**), lettuce (**B**), and basil (**C**) leaves after 2, 4, and 7 days of cultivation under four nitrogen levels (N80, N120, N150, and N180). Letters indicate significant differences among treatments (*p* < 0.05). Data is presented as the mean of twelve replicates (n = 12). Error bars indicate standard error.

Natural bacterial contamination of pak choi, lettuce, and basil leaves increased over time under all treatments (Figure 4). In pak choi, N180 showed significantly higher bacterial counts than all other nitrogen levels after 2 and 4 days (*p* < 0.05). No significant differences among treatments were observed at 7 DPIs. These results indicate that the highest nitrogen level (N180) promoted greater bacterial contamination compared with lower nitrogen levels.

In lettuce (Figure 4B), after 4 days, the N150 treatment had significantly higher bacterial concentrations than N80 and N120. No significant differences among treatments were observed after 2 and 7 days.

In basil leaves (Figure 4C), N150 had significantly lower bacterial concentrations than N80 after 2 and 4 days. After 7 days, the N120 treatment resulted in the greatest bacterial levels in basil leaves (though not significantly different from N80), whereas N150 had the lowest contamination (not significantly different from N180).

**Figure 5:**
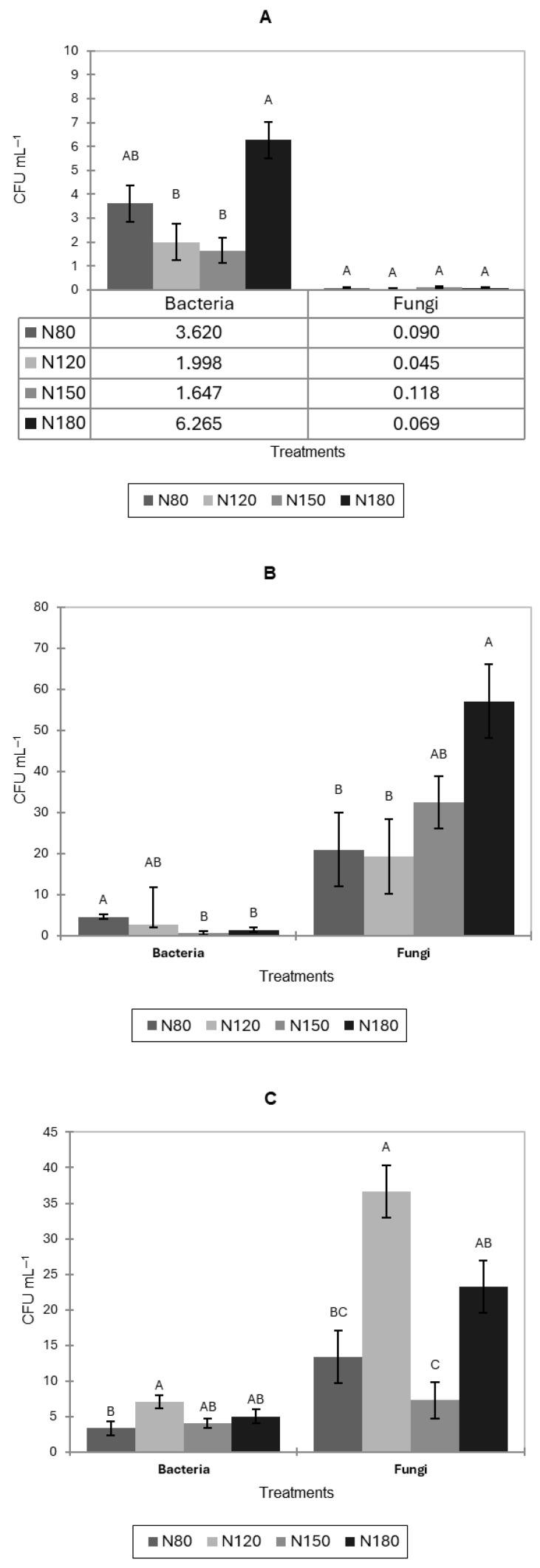
Natural bacterial and fungal contamination (CFU mL^−1^) on leaves of pak choi (**A**), lettuce (**B**), and basil (**C**) after postharvest storage under four nitrogen levels (N80, N120, N150, and N180) after 2 days of cultivation. Letters indicate significant differences among treatments (*p* < 0.05). Data is presented as the mean of twelve replicates (n = 12). Error bars indicate standard error.

In pak choi, natural bacterial and fungal contamination in leaves at the end of postharvest storage varied across nitrogen treatments (Figure 5A). The highest bacterial contamination was observed under the N180 treatment, which was significantly greater than that in N120 and N150. The N80 treatment showed intermediate bacterial levels that did not differ significantly from N180. Fungal contamination levels were low across all treatments with no significant differences among nitrogen treatments (*p* > 0.05).

For lettuce (Figure 5B), the N80 treatment showed the highest bacterial level, significantly greater than N150 and N180, while N120 did not differ significantly from N80. Fungal contamination increased substantially across treatments. The highest fungal level was observed under N180, which was significantly greater than N80 and N120. N150 showed an intermediate fungal concentration that did not differ significantly from other treatments.

The highest bacterial contamination of basil occurred under the N120 treatment, which was significantly greater than N80 (Figure 5C). Treatments N150 and N180 showed intermediate bacterial levels that did not differ significantly from either N80 or N120. Fungal contamination levels showed notable variation among treatments. The N120 treatment exhibited the highest fungal count, significantly exceeding those of N80 and N150. The lowest fungal concentration was observed in N150, which was significantly lower than in N120 and N180. N80 displayed intermediate fungal levels that did not differ significantly from the N150 and N180.

### 3.3. Incidence of B. cinerea on Leafy Vegetables Leaves During Posthavest Storage at 22 °C

**Figure 6:**
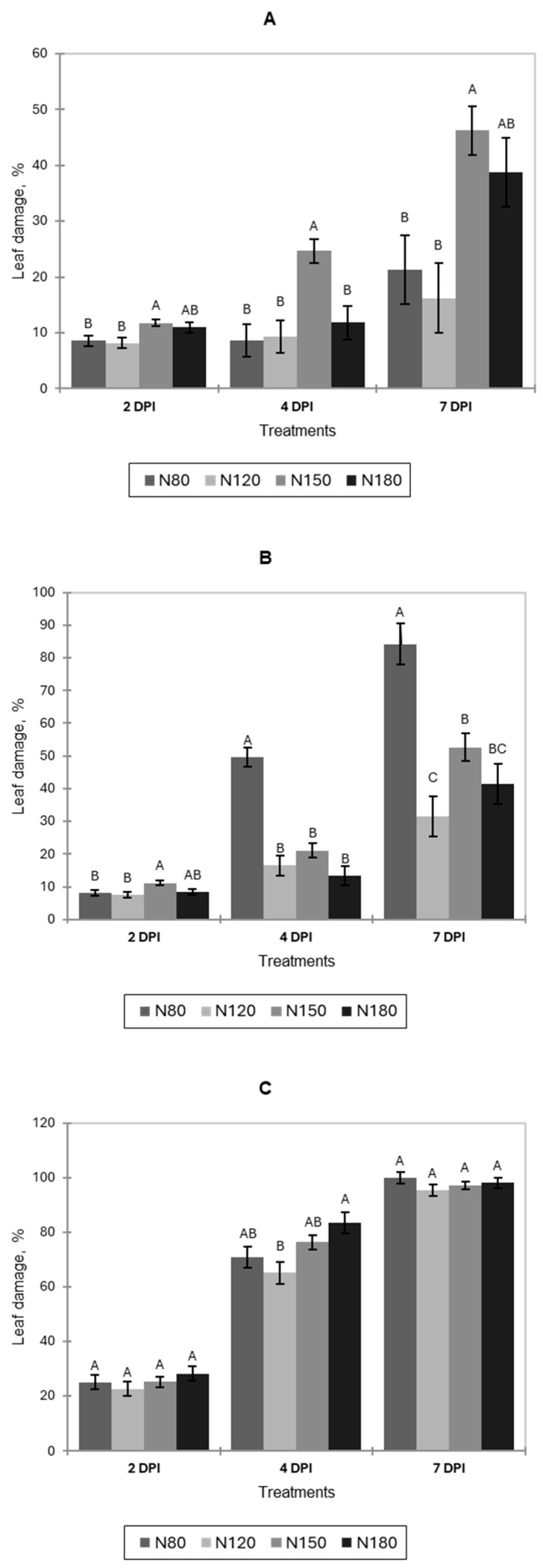
Incidence of *B. cinerea* on leaves of pak choi (**A**), lettuce (**B**), and basil (**C**) during postharvest storage at 22 °C, expressed as a percentage of leaf damage at 2, 4, and 7 days post-inoculation (DPI) under four nitrogen levels (N80, N120, N150, and N180). Letters indicate significant differences among treatments (*p* < 0.05). Data is presented as the mean of twelve replicates (n = 12). Error bars indicate standard error.

The incidence of artificial infection with *B. cinerea* on pak choi leaves increased progressively during postharvest storage at 22 °C across all nitrogen treatments (Figure 6A). At 2 days post-inoculation (DPIs), leaf damage was low (around 8–12%) in all treatments. N150 showed slightly higher infection and differed significantly from N80 and N120, but not from N180. By 4 DPIs, disease severity rose markedly, with N150 remaining the highest and significantly greater than the other treatments. By 7 DPI, severity increased further; N150 again exhibited the greatest damage, significantly higher than N80 and N120. N180 did not differ statistically from N80, N120, or N180. Overall, *B. cinerea* intensified over time, and nitrogen supply influenced disease development, with N150 consistently producing the highest damage relative to the other nitrogen levels.

In lettuce, leaf damage at 2 DPIs was low (<15%) for all treatments (Figure 6B). N150 showed slightly higher infection and differed significantly from N80 and N120, but not from N180. By 4 DPI, severity increased substantially. N80 had the highest damage, significantly greater than N120, N150, and N180, which did not differ from one another. The same pattern held at 7 DPI: N80 again showed the highest severity and differed significantly from N120, N150, and N180. Additionally, N120 was significantly lower than N150 and N180, which did not differ.

For basil, no significant differences among treatments were observed at 2 DPIs (Figure 6C). At 4 DPIs, N120 showed the lowest infection and differed significantly from N180, whereas N80 and N150 did not differ from any treatment. At 7 DPI, leaf damage exceeded 90% in all treatments, with no significant differences among nitrogen levels.

### 3.4. Incidence of B. cinerea on Leafy Vegetables Leaves During Postharvest Storage at 4 °C

**Figure 7:**
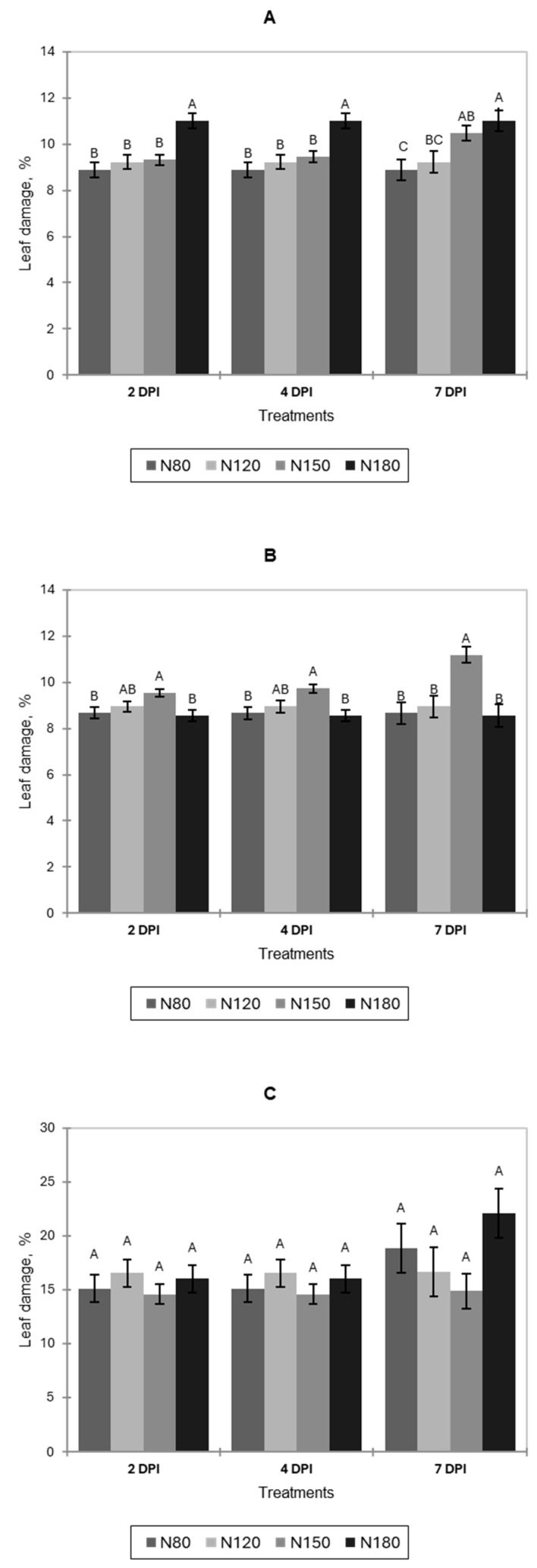
Incidence of *B. cinerea* on leaves of pak choi (**A**), lettuce (**B**), and basil (**C**) during postharvest storage at 4 °C, expressed as a percentage of leaf damage at 2, 4, and 7 days post-inoculation (DPI) under four nitrogen levels (N80, N120, N150, N180). Letters indicate significant differences among treatments (*p* < 0.05). Data is presented as the mean of twelve replicates (n = 12). Error bars indicate standard error.

The incidence of artificial infection with *B. cinerea* progressed slowly on pak choi, lettuce, and basil during postharvest storage at 4 °C, with modest but consistent increases in leaf damage across all nitrogen treatments (Figure 7). In pak choi, at 2 DPIs, damage was ~9–11% (Figure 7A). N180 was the highest and differed significantly from N80, N120, and N150, which did not differ from one another. Similar results were observed at 4 DPIs. At 7 DPIs, treatment separation widened: N80 < N120 < N150 < N180. N180 remained significantly greater than other treatments but did not differ compared to N150. The significantly lower leaf damage was N80, but it did not differ from N120.

For lettuce, at 2 DPIs, damage was ~8–10% (Figure 7B). N150 was the highest and significantly differed from N80 and N180, while N120 was intermediate. The same tendency was observed at 4 DPIs. Similarly, at 7 DPIs, N150 was significantly higher than N80, N120, and N180, which did not significantly differ from each other.

No significant difference was observed between nitrogen treatments and DPI for basil (Figure 7C).

Overall, compared to postharvest storage at 22 °C, refrigeration limited disease development.

## 4. Discussion

Our results demonstrate that N availability exerts a significant influence on both the phyllosphere microbial loads and the postharvest development of gray mold (*B. cinerea*) in leafy vegetables. This study quantified phyllosphere microbes using culture-based CFU counts on PDA. These media and plate-count methods recover only the culturable fraction of the community and do not capture unculturable taxa, absolute richness, or fine-scale changes in community composition. Therefore, the CFU results should be interpreted as changes in culturable load rather than as a full description of phyllosphere diversity or composition. Culture-independent (amplicon or metagenomic) sequencing is needed to define the full phyllosphere microbiome and confirm whether observed CFU changes reflect broader community shifts. Lettuce grown with higher nitrogen inputs exhibited markedly elevated microbial loads—both in the hydroponic solution and on leaf surfaces—indicating that surplus N can facilitate fungal persistence on harvested tissues. In contrast, pak choi maintained consistently low natural microbial contamination across all N treatments, with only a bacterial rise at N180. This suggests that pak choi may possess intrinsic resistance traits (such as antimicrobial compounds or slower tissue senescence) that limit microbial buildup regardless of fertilization level. Also, it suggests that lettuce creates a more microbe-favorable milieu under high N (perhaps via root exudates or shedding of organic matter), while pak choi inherently resists microbial growth in its immediate environment. Intriguingly, basil showed a non-linear response in natural microbial contamination. Leaves from an intermediate N supply (N120) had the greatest total microbial load (for both bacteria and fungi) after storage—higher than either N-deficient or N-excess conditions. In fact, the N150 treatment often yielded among the lowest microbial counts in basil, significantly lower than those at N120 in both bacterial and fungal assays. Such a U-shaped pattern implies that very low or very high N might impose stress (nutrient deficiency or excess) that indirectly constrains microbial growth on basil, whereas a moderate N level creates optimal conditions for epiphytic microbes to proliferate. Overall, these findings confirm our first hypothesis that elevated N can stimulate phyllosphere microbial proliferation—especially for a highly responsive host like lettuce—while also revealing that certain leafy vegetables (pak choi) can inherently suppress phyllosphere microbiota proliferation even under rich N conditions, and others (like basil) display non-linear, species-specific responses.

At ambient temperature (22 °C), N supply also strongly modulated gray mold severity, with distinct trends in each crop. In pak choi, disease severity rose with higher N: leaves from N150 plants consistently developed the largest lesions at each time point. At 2 DPIs, for instance, pak choi leaves from N150 had a higher infection rate (about 10–12% leaf area), which was significantly greater than in low-N and moderate-N plants (N80 and N120), though not different from the N180 treatment. By 4 DPIs, lesions expanded markedly on all pak choi, but remained most severe in N150 leaves, which had significantly more gray mold than all other N levels. By 7 DPIs, infection had progressed further; the N150 treatment again exhibited the greatest damaged area, significantly higher than in N80 and N120. Leaves from the highest N treatment (N180) also tended toward high levels of disease (roughly comparable to N150 by 7 DPIs), although, statistically, N180 did not differ from the other treatments at that final time. Overall, pak choi grown with elevated N (especially the N150 and N180 regimes) suffered roughly 1.5–2 times more gray mold by one week than pak choi grown with low N (N80). This indicates that excessive N fertilization predisposed pak choi tissues to faster and more extensive colonization by the pathogen, possibly by reducing structural integrity or diluting defense compounds in the leaves.

Lettuce displayed a more complex, time-dependent response. In the early infection stage (2 DPIs), we observed slightly more gray mold in the high-N treatments—notably, lettuce at N150 had greater lesion coverage than lettuce at N80 or N120—suggesting that N surplus can increase initial susceptibility in lettuce. By 4 DPIs, this pattern reversed: the low-N lettuce (N80) developed the highest disease severity, with lesion areas approximately double those on any of the higher-N lettuce samples. At this mid-point, N80 leaves were significantly more diseased than the leaves from N120, N150, or N180 (which showed similar, lower levels of infection). By 7 DPIs, low-N lettuce continued to fare the worst: N80 plants sustained the greatest gray mold damage (nearly twice the diseased area of some higher-N treatments). In contrast, the moderate-N lettuce (N120) maintained the lowest disease severity by one week, significantly less than that in the high-N treatments. The high-N lettuce groups (N150 and N180) showed intermediate levels of infection at 7 DPIs—considerably lower than the low-N lettuce, but slightly higher than the N120 plants. These temporal dynamics underscore that both N deficiency and N excess can be detrimental for lettuce, but at different stages: an ample N supply appears to confer some resistance advantage as storage progresses, whereas N deprivation leads to progressively worsening decay. In short, lettuce with balanced nutrition (around N120) was most effective at limiting gray mold over the course of a week, while lettuce at the extreme N levels (very low or very high) became more susceptible at one or another point in time.

Basil’s response at 22 °C also shifted over time, reflecting an optimal N level in the short term but eventually overwhelming susceptibility. In the early stages, all basil leaves were moderately infected, and no significant N-related differences were evident at 2 DPIs. By 4 DPIs, however, the intermediate-N basil (N120) had developed the smallest lesions, indicating the least disease progression. The lesion size at N120 was significantly lower than that on the highest-N basil (N180), which suffered the most infection by 4 DPIs. The N80 and N150 basil plants showed intermediate gray mold severity at that time—not significantly different from either the best or the worst treatments—suggesting that the benefits of N120 were somewhat specific in comparison to the extreme N regimes. Nevertheless, by 7 DPIs, all basil leaves were extensively infected, regardless of N supply, with lesions covering well over 90% of leaf area in every treatment, and no statistical differences among N levels. Basil’s extreme susceptibility to *B. cinerea* eventually overrode any nutritional advantage, meaning that even the N120 plants (which initially had the least disease) were fully colonized by the end of the storage period. In summary, under warm conditions, the general trend was that higher N often aggravated gray mold severity (as seen consistently in pak choi and in the later stages for lettuce and basil), whereas moderate N sometimes minimized disease (particularly in the early-to-mid storage period for lettuce and basil). These dynamics underscore that the relationship between N nutrition and disease is not strictly linear; plant nutritional status can influence not only the magnitude but also the timing of pathogen development on postharvest tissues.

Refrigerated storage at 4 °C dramatically suppressed the incidence of *B. cinerea* in all three crops, as expected, yet some N-driven differences still manifested under these refrigerated conditions. *B. cinerea* grows optimally at around 15–20 °C and becomes nearly dormant at near-freezing temperatures. Accordingly, in our study, the infection progressed much more slowly at 4 °C than at 22 °C for every N treatment, reflecting the well-known effect of low temperature in hindering fungal growth and sporulation. After 7 days at 4 °C, lettuce and pak choi leaves inoculated with *B. cinerea* showed only minimal lesion development (just a few percent of leaf area on average), whereas at 22 °C, those lesions had expanded to tens of percent. This highlights refrigeration as an effective short-term strategy to keep gray mold in check for produce that tolerates cold. Even so, higher-N levels did lead to significantly more gray mold damage in certain cases during refrigerated storage. In pak choi at 4 °C, a nitrogen effect reappeared by the end of the week: leaves from the high-N treatments (particularly N180) had accumulated slightly greater lesion areas than those from lower N. For example, by 7 DPIs, pak choi grown with excess N had roughly 20–30% more diseased leaf area than pak choi grown with the lowest N, a smaller but still notable difference that mirrors the N effect observed at ambient temperature. Lettuce also showed a subtle N-related pattern under refrigeration. Although all N treatments kept gray mold to very low levels in lettuce at 4 °C, the N150 treatment consistently yielded the highest (albeit still small) amount of disease. By 7 DPIs, N150 lettuce had a significantly greater infected area than lettuce at any other N level (N80, N120, or N180), which all remained statistically alike to each other. This result suggests that even under refrigerated conditions, an intermediate-high N supply (around 150 mg L^−1^ in this case) may slightly compromise lettuce’s resistance relative to both a balanced N regime and even an extreme N surplus (N180).

By contrast, basil derived essentially no benefit from refrigeration. Despite the refrigerated temperature slowing fungal growth, basil leaves at 4 °C developed severe gray mold in the days following inoculation, and by one week, the basil in all N treatments was nearly as decayed as at ambient conditions. No significant differences were detected among N treatments for basil at 4 °C—all were almost completely rotted by 7 DPIs. Basil’s consistently high vulnerability to gray mold under refrigerated storage was striking at 4 °C; basil leaves (regardless of N supply) became covered in *Botrytis*, whereas lettuce and pak choi leaves at 4 °C still had only small, contained lesions. Basil leaves suffer chilling injury at temperatures below 10–12 °C, leading to necrosis and decay; although refrigeration slows *Botrytis*, the cold stress makes basil more vulnerable, reflecting a documented trade-off between pathogen suppression and tissue integrity [22,23]. Indeed, the literature reports that storing basil at 5 °C causes severe chilling injury and makes the tissue prone to rapid decay [22,23]. Thus, in our refrigerated-storage experiment, basil likely experienced physiological stress (membrane damage and tissue necrosis) due to the 4 °C condition, effectively compromising its defenses and enabling *B. cinerea* to colonize even in the refrigerated conditions. In contrast, lettuce and pak choi tolerated low temperatures much better; their tissues remained intact at 4 °C and were therefore less hospitable to the pathogen. This species-specific difference is crucial for postharvest handling: what is “refrigerated” for one crop (beneficially slowing pathogens) may be “freezing” for another (predisposing it to pathogen attack). Lettuce and pak choi can be stored near 0 °C to virtually halt *B. cinerea* growth, but basil requires alternative postharvest strategies. For example, mild heat pre-treatments or storage at a relatively higher temperature (around 12 °C) with high humidity can be used to manage gray mold in basil, since conventional refrigeration would injure it and invite infection. In short, proper refrigerated-chain management is crucial to control *B. cinerea* in refrigerated-tolerant greens, while special handling is needed for refrigerated-sensitive herbs like basil.

These findings align with prior studies on N and disease. Excessive N has been linked to higher susceptibility to foliar pathogens and gray mold [24,25,26]. In our study, pak choi and lettuce at the highest N suffered more gray mold at harvest, consistent with reports that moderate N often reduces gray mold in crops (e.g., in strawberry, lettuce, grapevine [25,26]) and that *Arabidopsis* at low nitrate concentrations was less susceptible to *B. cinerea* than at high nitrate concentrations [24]. Notably, our pak choi was grown with nitrate-based N (common in hydroponics), which may partly explain its vulnerability, since nitrate nutrition tends to increase *B. cinerea* infection relative to ammonium [24]. Likewise, legumes and other plants often show reduced foliar disease under adequate-but-not-excess N [24]. Thus, a broad consensus is emerging that judicious N management can suppress many pathogens—a principle reinforced by our data on postharvest leaf pathogens. However, the N–disease relationship can be complex and crop-specific. Some studies have documented opposite trends under conditions or with different host–pathogen systems. A prime example is tomato gray mold: Hoffland et al. [27] and Lecompte et al. [28] reported that *B. cinerea* caused more severe disease in tomato plants under N deficiency, whereas higher N levels improved resistance in that case. Our findings in lettuce mirror this nuance—at certain time points, low-N lettuce was actually more diseased than high-N lettuce (notably, we observed significantly larger lesions in N80 lettuce than in N180 lettuce at 4 DPI under 22 °C). This suggests that nitrogen deprivation can also weaken plant defenses (perhaps by causing poor nutritional status and stress) and can thereby increase susceptibility, an observation supported by earlier work on other pathogens (e.g., powdery mildew severity can increase in N-starved cereal plants according to Snoeijers et al. [29]. In our lettuce, the fact that moderate N (N120) consistently minimized *B. cinerea* damage relative to both N extremes implies an optimal nutrition zone for defense. Too little N may limit the plant’s ability to mount defenses (e.g., producing antifungal proteins or healing wound sites), while too much N is likely to create a permissive environment for the pathogen. The U-shaped response curve we saw in basil (where intermediate N was best for lowest decay) further exemplifies this principle. These findings resonate with the notion in plant pathology that the effect of N on disease is not linear but can be optimal at intermediate levels. Factors like the form of N (nitrate vs. ammonium), the timing of N uptake, and the specific host metabolism all modulate the outcome [24]. Our data across three species highlight that each plant pathogen system has its own “sweet spot” of nutrition for resistance. Therefore, blanket statements about “high N = more disease” do not universally apply; instead, the interaction must be evaluated case by case.

Nitrogen fertilization also clearly influenced the phyllosphere community and disease outcomes. Studies in rice have shown that very high N input (e.g., 330 kg ha^−1^) enriches foliar fungal pathogens, whereas moderate N (around 210 kg ha^−1^) supports more beneficial microbes and fewer pathogens [30]. We observed a similar pattern in our work: lettuce grown with excessive N (N180) had far more fungal colonies on its leaves and more severe gray mold, while moderate-N lettuce had much lower fungal contamination and disease severity. This trend is corroborated by other research noting that over-fertilization increases the abundance of leaf pathogens [31]. Likewise, a recent study found that low-N conditions allowed a beneficial bacterium (*Massilia*) to flourish on rice leaves and protect against blast fungus, a benefit lost under high N [32]. In summary, balanced or lower N levels tend to foster a protective phyllosphere community and stronger plant defenses, whereas excessive N tilts the balance in favor of foliar pathogens.

Our findings suggest practical strategies for reducing postharvest decay by managing N. Growers should avoid high N applications: supplying just enough N for optimal growth can significantly lower pathogen loads on leaves and improve plants’ innate decay resistance, while also reducing fertilizer waste and pollution. Complementary approaches include harnessing the phyllosphere microbiome: for example, applying beneficial biocontrol microbes to foliage may help suppress pathogens when plants are not over-fertilized. Importantly, pre- and postharvest practices should be integrated. Fresh leafy greens like lettuce and pak choi should be cooled quickly (near 0 °C) to halt *B. cinerea*, whereas herbs like basil require milder storage (about 12 °C with high humidity) to avoid chilling injury. In short, a combined strategy of moderate N nutrition, an enhanced beneficial microbial community, and appropriate storage conditions can minimize postharvest decay without relying on chemical fungicides.

This study focused on broad microbial counts and a single pathogen, so future work should extend these findings. Phyllosphere sequencing (e.g., 16S/ITS amplicon or metagenomic analysis) could identify which microbial taxa increase or decrease with N to pinpoint protective or harmful species. It would also be valuable to test whether the effects of N on disease hold for other common postharvest pathogens (such as bacterial soft rots) and under field or commercial conditions. Measuring plant tissue nutrient levels and defense metabolites under different N regimes could further clarify the mechanisms of N-linked susceptibility. Additionally, exploring different nitrogen forms (nitrate vs. ammonium) and deliberately introducing known beneficial microbes to high-N plants are promising practical directions. In summary, refining nitrogen management and leveraging the plant microbiome represent important steps toward reducing postharvest diseases in an eco-friendly way.

## 5. Conclusions

Nitrogen fertilization had clear, crop-specific effects on phyllosphere microbes and postharvest gray mold severity. In lettuce, high N supply (180 mg L^−1^) elevated culturable microbial counts in the growing solution and on leaves, but plants under low N (80 mg L^−1^) developed the most extensive *B. cinerea* lesions after inoculation. Lettuce grown with moderate N (120–150 mg L^−1^) had a lower incidence, indicating that balanced N nutrition minimizes gray mold. Pak choi maintained low microbial contamination at all N levels, yet gray mold severity increased consistently with higher N: leaves from high-N plants (150–180 mg L^−1^) sustained roughly double the damage of low-N (80 mg L^−1^) plants. Basil exhibited a non-monotonic microbial response, with the greatest bacterial and fungal loads at an intermediate N level (120 mg L^−1^), but it was uniformly vulnerable to gray mold. By one week at 22 °C after artificial inoculation with *B. cinerea*, basil leaves were nearly completely infected regardless of N regime, and any early benefit of moderate N (slower infection at day 4) had disappeared by day 7.

Storage temperature modulated these effects. Refrigeration (4 °C) greatly suppressed gray mold progression in lettuce and pak choi, although even under cold storage, high-N pak choi still decayed more than low-N by the end of the week. In contrast, basil leaves suffer severe chilling injury and decay at 4 °C, independent of N level. These findings underscore the importance of tailored N management and handling for leafy crops: avoiding excessive N in pak choi, maintaining balanced N in lettuce, and emphasizing non-chilling postharvest conditions for basil can reduce microbial buildup and postharvest losses.

## Figures and Tables

**Table 1:** Composition of nutrient solutions used in experiments.

Treatments	The Concentrations of Mineral Nutrients, mg L^−1^										
	N	P	K	Ca	Mg	S	Fe	Mn	Cu	B	Zn
N80	80	25	100	80	40	53	2	0.1	0.1	0.2	0.1
N120	120										
N150	150										
N180	180										

## Data Availability

The original contributions presented in this study are included in the article. Further inquiries can be directed to the corresponding author.

## Abbreviations

The following abbreviations are used in this manuscript:

DPIs	Days post-infection
N80	Nitrogen 80 mg L^−1^
N120	Nitrogen 120 mg L^−1^
N150	Nitrogen 150 mg L^−1^
N180	Nitrogen 180 mg L^−1^
